# The Honey Bee Pathosphere of Mongolia: European Viruses in Central Asia

**DOI:** 10.1371/journal.pone.0151164

**Published:** 2016-03-09

**Authors:** Khaliunaa Tsevegmid, Peter Neumann, Orlando Yañez

**Affiliations:** 1 Institute of Bee Health, Vetsuisse Faculty, University of Bern, Bern, Switzerland; 2 Mongolian State University of Agriculture, Ulaanbaatar, Mongolia; 3 Agroscope, Swiss Bee Research Centre, Bern, Switzerland; 4 Bee Protection Laboratory, Faculty of Science, Chiang Mai University, Chiang Mai, Thailand; Goethe University Frankfurt, GERMANY

## Abstract

Parasites and pathogens are apparent key factors for the detrimental health of managed European honey bee subspecies, *Apis mellifera*. Apicultural trade is arguably the main factor for the almost global distribution of most honey bee diseases, thereby increasing chances for multiple infestations/infections of regions, apiaries, colonies and even individual bees. This imposes difficulties to evaluate the effects of pathogens in isolation, thereby creating demand to survey remote areas. Here, we conducted the first comprehensive survey for 14 honey bee pathogens in Mongolia (N = 3 regions, N = 9 locations, N = 151 colonies), where honey bee colonies depend on humans to overwinter. In Mongolia, honey bees, *Apis* spp., are not native and colonies of European *A*. *mellifera* subspecies have been introduced ~60 years ago. Despite the high detection power and large sample size across Mongolian regions with beekeeping, the mite *Acarapis woodi*, the bacteria *Melissococcus plutonius* and *Paenibacillus larvae*, the microsporidian *Nosema apis*, Acute bee paralysis virus, Kashmir bee virus, Israeli acute paralysis virus and Lake Sinai virus strain 2 were not detected, suggesting that they are either very rare or absent. The mite *Varroa destructor*, *Nosema ceranae* and four viruses (Sacbrood virus, Black queen cell virus, Deformed wing virus (DWV) and Chronic bee paralysis virus) were found with different prevalence. Despite the positive correlation between the prevalence of *V*. *destructor* mites and DWV, some areas had only mites, but not DWV, which is most likely due to the exceptional isolation of apiaries (up to 600 km). Phylogenetic analyses of the detected viruses reveal their clustering and European origin, thereby supporting the role of trade for pathogen spread and the isolation of Mongolia from South-Asian countries. In conclusion, this survey reveals the distinctive honey bee pathosphere of Mongolia, which offers opportunities for exciting future research.

## Introduction

Despite the importance of managed colonies of European honey bee subspecies, *Apis mellifera*, for pollination [1], only their sudden losses reported in autumn 2006 and spring 2007 in the USA have brought their health into focus [2], receiving now considerable public and political attention [3].

Acute poisoning by conventional and novel insecticides, habitat fragmentation, poor bee management, parasites and pathogens, such as *Nosema* spp. [4], *Paenibacillus larvae* [5] and in particular the ectoparasitic mite *Varroa destructor* [6] and the lethal virus epidemics it initiates and vectors [7–8] have all been proposed as possible causal agents for compromised honey bee health or colony death [9–10]. Recent analyses of long-term surveys of honey bee colony losses in 16 countries point to parasites and diseases as the single most important cause of honey bee colony losses [11].

Apicultural trade appears to be the major driving force behind the almost global distribution of most honey bee parasites and pathogens [12–13] and, thereby increasing the probability of multiple infestations/infections of regions, apiaries, colonies and even individual bees [14–15]. This imposes difficulties to evaluate the effects of pathogens in isolation, thereby creating demand to survey remote areas. Here, we conducted the first comprehensive survey for 14 honey bee pathogens and determine their prevalence in Mongolia (N = 151 colonies), where this pollinator is not native, but has been introduced in 1959 [16]. Mongolia is a very large country, which is surrounded by natural geographical barriers such as the Gobi desert and the Altai mountains. Moreover, mean temperatures are extremely low in winter (from -16 to -32°C) [17]. Mongolia is the least densely populated country globally, thereby creating exceptional isolation between apiaries (up to 600 km). Taken together with the lack of native honey bees and the natural barriers surrounding Mongolia, this isolation and the harsh climate are likely to result in a unique honey bee pathosphere probably reflecting the past import of European stock rather than input from neighboring countries. Here we compare the Mongolian honey bee pathosphere with ones from other European and Asian countries. Moreover, we tested the isolation hypothesis using phylogenetic analyses of the detected viruses to identify their possible origins. If the isolation actually occurred, Mongolian bee viruses should be more related to viruses from European origin than from neighboring South-Asian countries.

## Material and Methods

### Sampling

**Fig 1:**
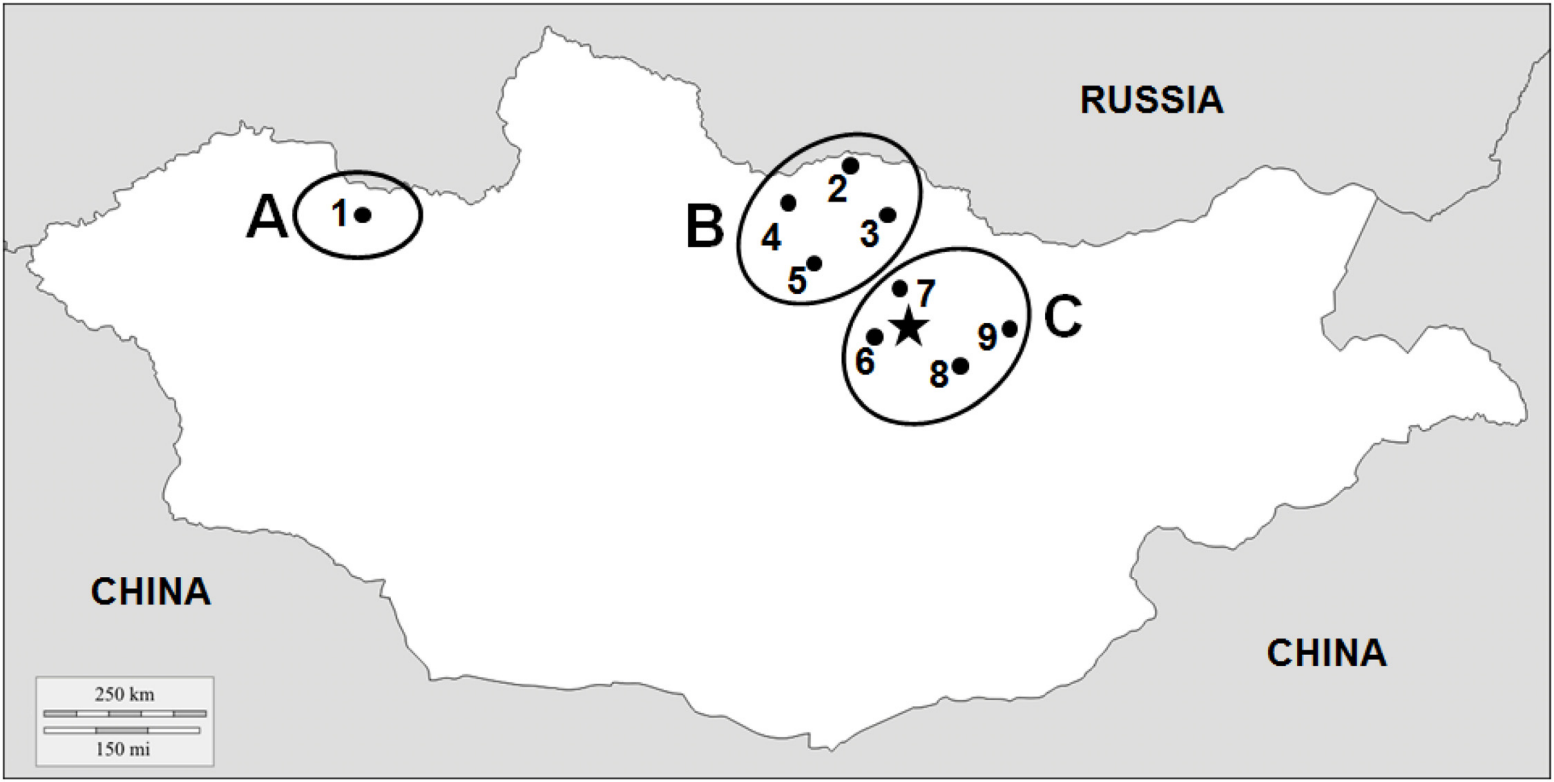
Sampling locations in Mongolia (• 1 to 9 = locations; ○ = regions A, B, and C; ★ = Ulaanbaatar).

Permissions for sampling the honey bee colonies were obtained from the local land owners and beekeepers and the study did not involve the collection and/or manipulation of endangered or protected species. In summer 2013 (July—August), adult honey bee workers were sampled from the brood nests of 151 queenright colonies (N = ~100 each) at nine locations (comprising 1–3 apiaries each) from three different regions across Mongolia (Fig 1). One sampling location (N = 15 colonies from two apiaries) is in the west region (= A), where beekeeping is very rare. This region is characterized by high mountains and very harsh winters (multiyear average temperature in January -32.2°C [17]). From six colonies of one apiary in region A, ten sealed drone brood cells were also taken. In the north of Mongolia is the region (B) with sampling locations 2–5 (N = 91 colonies from 12 apiaries), where apiculture is more intense due to advantageous local conditions (abundant nectar flows and warmer temperature during summer). Four locations (6–9, N = 45 colonies from 7 apiaries) were sampled in the central region (C) around the capital Ulaanbaatar, which is characterized by a high elevation of the sampling sites (~1200 m above sea level) and comparatively advanced beekeeping practices. Average temperature for the last 15 years in January was -18.9 and -18.2°C for regions B and C, respectively [17]. Beekeepers from all locations reported that they did not treat against Nosemosis. Similarly, beekeepers from locations 1 (region A) and 6 (region C) also did not treat against varroa mites. In contrast, beekeepers from region B and location 7 (region C) did regularly use acaricides such as amitraz or fluvalinate. The use of antibiotics was not reported by beekeepers from all regions. Sampling was conducted in areas that not require permission from national authorities. All samples were kept on ice during transportation to the laboratory and stored at -80°C until molecular analysis.

### *Varroa destructor* assessment

Estimates of phoretic mite infestation rates were obtained from all colonies by washing the adult worker samples according to standard procedures [18]. In six colonies from region A (see above), brood mites were also obtained by dissecting the sealed drone brood cells [18].

### Sample homogenization

Fifty adult workers from each sampled colony were pooled for homogenization [19]. It was done in two steps: 1) preliminar dissociation of tissues in 10 ml TN buffer (0.1 M Tris-HCl, 0.1 M NaCl, pH 7,6) using MACS M tubes and a Dispomix^®^ Drive homogenizer (Medic tools), 2) 1 ml aliquot from each sample was fully homogenized in a Mixer Mill MM 300 (RETSCH GmbH) with 3 mm steel beads. For *V*. *destructor*, individual mites were directly homogenized using a Mixer Mill MM 300 (RETSCH GmbH) machine in 100 μl TN buffer with 3 mm metal beads.

### DNA extraction

From pooled worker samples, DNA was extracted from 50 μl of the sample homogenate using the NucleoSpin^®^ 96 Tissue kit (Machery-Nagel) by following the supplier’s guidelines. DNA was eluted in 60 μl elution buffer (5 mM Tris/HCl, pH 8.5). From *V*. *destructor*, DNA was extracted using 100 μl of Chelex^™^ 5% solution per mite (N = 13) and incubation for 20 min at 95°C [20]. For each sample, DNA was 10-fold dilute using RNAse-free water.

### RNA extraction and reverse transcription

RNA was extracted from pooled worker samples and single *V*. *destructor* mites (N = 10) using 50 μl of the sample homogenates and NucleoSpin^®^ RNA kit (Macherey-Nagel) with final elution in 50 μl of RNAse-free water following the manufacture’s guidelines. Reverse transcription was performed in ~1 and ~0.2 μg of extracted RNA from pooled worker samples and mites, respectively, using the M-LV RT enzyme (Promega) with 100 μM of random hexamers in 25 μl final volume following the manufacturer’s recommendations. Before PCR amplifications, the resulting cDNAs were diluted 10-fold using RNAse-free water.

### PCR assays

The detection of honey bee pathogens was performed by PCR assays. For all these molecular analyses, respective positive and negative controls were included. The primers used are listed in S1 Table. All PCR products were analyzed using 1.2% agarose gel electrophoreses and visualized using an UV transilluminator.

To evaluate the *V*. *destructor* haplotypes [21], one mite was individually analyzed from infested colonies (N = 13) from all three regions. mtDNA was amplified using published COI primers [21]. Amplifications performed using KAPA 2G Fast Ready Mix+dye reagents in 25 μl volumes containing 3 μl template DNA, 12.5 μl of 2X Reaction Buffer and 0.25 μl of each forward and reverse primers (10 μM). PCR conditions were 2 min initial incubation at 95°C, 35 cycles of 20 sec at 95°C for denaturation, 20 sec at 55°C for annealing, 30 sec at 72°C extension and a final step of 2 min at 72°C.

*Acarapis woodi*, *Nosema apis* and *N*. *ceranae* were tested using previously reported specific primers and respective PCR conditions (S1 Table) [22–23] using MyTaq kit (Bioline) following the supplier’s guidelines. For the detection of *Melissococcus plutonius* and *Paenibacillus larvae* PCR were performed using 2 μl of 10-fold diluted genomic DNA using MyTaq (Bioline) reagents in 25 μl final volume reaction containing 2.5 μl of 10X Reaction Buffer, 1 μl of dNTPs Mix (10 mM), 1 μl of each forward and reverse primer (10 μM) and 0.125 μl of Taq polymerase (1.25 U). For the detection of *M*. *plutonius* and *P*. *larvae*, primer sets for the Sod-A and the 16S ribosomal RNA genes were used, respectively (S1 Table). PCR conditions for both bacteria were as follows: initial denaturation at 95°C for 2 min, 35 cycles of denaturation at 95°C for 20 sec, annealing at 56°C for 20 sec, at 72°C for 30 sec and extension at 72°C for 2 min.

Amplifications for the detection of viruses were performed with 2 μl diluted cDNA using MyTaq (Bioline) reagents and PCR conditions as describe above. Positive samples for Sacbrood virus (SBV), Black queen cell virus (BQCV), DWV, and Chronic bee paralysis virus (CBPV) were sequenced using different primers (S1 Table). PCR was performed using HIFI Kapa kit reagents in 25 μl volumes containing 2 μl template cDNA, 5 μl of 5X Reaction Buffer, 0.75 μl of dNTPs Mix (10 mM), 0.75 μl of each forward and reverse primers (10 μM) and 0.5 μl of Taq polymerase. The reaction condition was 95°C for incubation 2 min, 98°C for denaturation 20 sec., 57°C for annealing 20 sec, 72°C for extension 30 sec and 2 min for a final step at 72°C.

### *Nosema* spp. spore quantification

*Nosema* spp. spore counts were performed in PCR-positive colonies (N = 14). Individual bees (N = 50 for each colony) were crushed with a mortar in 1000 μl water [24] and then spores were counted in a small droplet using a haemocytometer and a light microscope (x400) [25] calculating spores per bee.

### Phylogenetic analyses

Phylogenetic analyses of the detected viruses were performed to confirm their species status and to detect their geographic affiliation. The identity of the detected species was confirmed using the NCBI Blast searches in Genbank (https://blast.ncbi.nlm.nih.gov). To infer the phylogenetic trees, the nucleotide sequences of the samples and of other annotated sequences retrieved from Genbank were aligned using the MUSCLE program and compared using the maximum likelihood method with the MEGA5.2 program under the Tamura 3-parameter [26]. The tree topology was evaluated by bootstrap resampling (1,000 times) [26]. Virus sequences were submitted to GenBank under accession numbers LN875562-LN875582.

### Statistical analyses

The prevalence of the honey bee pathogens was compared between geographical regions using Pearson Chi Square tests. One-way ANOVA was used to test difference in the number of *Nosema* spp. spores in individual workers between locations. A Pearson correlation was used to assess the association between the prevalence of *V*. *destructor* mites and DWV. The statistical analyses were performed using the NSCC 10 statistical software package.

## Results

### Mites

While *Acarapis woodi* was not detected in any sample, *Varroa destructor* was recorded in all regions (Table 1). There were no significant differences in *V*. *destructor* phoretic mite infestation rates (number of mites per 100 bees) between the regions (Region A: 5.87; Region B: 0.38; Region C: 5.96; Pearson Chi Square test, Chi Square = 5.2, df = 2, p = 0.073). However, mite prevalence was significantly different (Pearson Chi Square test, Chi Square = 22.3, df = 2, p<0.001). Region B was less infested by mites, 14.3% (13 out 91 colonies); followed by region C (37.77%; 17/45) and the highest mite infestation was found in region A (66.7%; 10/15). Colonies from all four locations of region B were infested with *V*. *destructor* (4.5–66.7%); three locations out of four of the region C were infested at 33.3–100% and location 7 was free from the mite. At region A, screening of sealed drone brood exhibited infestation of all six colonies, with up to 6 mites from a single cell. Regarding the *V*. *destructor* haplotype identity, the blast comparison of the 797-nt sequences, corresponding to the cox1 mitochondrial gene from 13 mites analyzed, showed 100% identity with the haplotype K1.

**Table 1:** Prevalence of detected pathogens in the three sampled regions. Prevalence of parasites and pathogens are given in percentages followed, in brackets, with the number of positive colonies and the total number of colonies screened, respectively.

Region	Location	Number of colonies	*Varroa destructor*	*Nosema ceranae*	SBV	BQCV	DWV	CBPV
A	1	15	66.67% (10/15)	0% (0/15)	27% (4/15)	0% (0/15)	0% (0/15)	0% (0/15)
		Overall A	66.67% (10/15)	0% (0/15)	27% (4/15)	0% (0/15)	0% (0/15)	0% (0/15)
B	2	17	35.29% (6/17)	11.8% (2/17)	100% (17/17)	47.1% (8/17)	41.2% (7/17)	0% (0/17)
	3	3	66.67% (2/3)	0% (0/3)	100% (3/3)	100% (3/3)	33.3% (1/3)	33.3% (1/3)
	4	27	11.11% (3/27)	44.44% (12/27)	100% (27/27)	100% (27/27)	3.7% (1/27)	15% (4/27)
	5	44	4.55% (2/44)	11.36% (5/44)	93.2% (41/44)	63.6% (28/44)	2.3% (1/44)	7% (3/44)
		Overall B	14.28% (13/91)	20.88% (19/91)	96.70% (88/91)	72.53% (66/91)	10.99% (10/91)	8.79% (8/91)
C	6	9	44.44% (4/9)	0% (0/9)	44% (4/9)	0% (0/9)	0% (0/9)	0% (0/9)
	7	15	0% (0/15)	0% (0/15)	20% (3/15)	0% (0/15)	0% (0/15)	0% (0/15)
	8	9	100% (9/9)	0% (0/9)	89% (8/9)	100% (9/9)	77.77% (7/9)	0% (0/9)
	9	12	33.33% (4/12)	0% (0/12)	33.3% (4/12)	0% (0/12)	0% (0/12)	8.3% (1/12)
		Overall C	37.77% (17/45)	0% (0/45)	42.22% (19/45)	20% (9/45)	15.55% (7/45)	2.22% (1/45)
**Overall**	9	151	26.49% (40/151)	12.58% (19/151)	73.5% (111/151)	49.66% (75/151)	11.25%(17/151)	5.96% (9/151)

### *Nosema* spp.

While *Nosema apis* was not detected at all, PCR-based detection revealed *N*. *ceranae* only in region B, with significant differences in prevalence between locations (Pearson Chi Square test, χ^2^ = 13.1, df = 3, p = 0.004). In the location 2, it was detected in 11.8% of the colonies (2/17), in the locations three, four and five 0% (0/3), 44.44% (12/27) and 11.36% (5/44), respectively. The number of spores in individual workers ranged from 5x10^4^ up to 30.4x10^6^ with significant differences between locations as determined by one-way ANOVA (F_(2,647)_ = 6.571, p = 0.001).

### Melissococcus plutonius, Paenibacillus larvae

All samples provided negative results for the casual agents of both European and American foulbrood by PCR-based detection.

### Viruses

Acute bee paralysis virus (ABPV), Kashmir bee virus (KBV), Israeli acute paralysis virus (IAPV), and Lake Sinai virus strain 2 (LSV-2) were not detected in this survey. Prevalence differed significantly among the four detected RNA viruses (Pearson Chi Square test, χ^2^ = 99.2, df = 3, p<0.001). SBV and BQCV were more prevalent (73.5% (111/151) and 49.6% (75/151), respectively) than DWV and CBPV (11.25% (17/151) and 5.96% (9/151), respectively).

#### Sacbrood virus

The SBV prevalence was significantly different between the regions (Pearson Chi Square test, χ^2^ = 64.7, df = 2, p<0.001). As shown in Table 1, region A displays 27% (4/15) detection of SBV. In the region B, SBV was abundant, with a prevalence of 100% in location 2 (17/17), location 3 (3/3) and 4 (27/27). Location 5 shows 93.2% (41/44) SBV positives. In the apiaries of four locations in the region C, the SBV prevalence ranged between 20–89% (3/15 and 8/9). The highest prevalence was found in location 8 (89%) and the lowest in location 7 (20%). The other two locations had a prevalence of 33.3% (4/12) and 44.4% (4/9), respectively.

#### Black queen cell virus

The BQCV prevalence was significantly different between regions (Pearson Chi Square test, χ^2^ = 49.7, df = 2, p<0.001). While BQCV was not detected in region A (0/15), all locations from region B tested positive with locations 3 and 4 being all positive (100% 3/3 and 100% 27/27). The samples of locations 2 and 5 show 47.1% (8/17) and 63.6% (28/44), respectively. Location 8 of the region C had 100% (9/9) infection with BQCV, but the other three locations were negative for this virus.

#### Deformed wing virus

This virus was less frequent compared to SBV and BQCV (Table 1). No significant difference in prevalence was found between regions (Pearson Chi Square test, χ^2^ = 2.7, df = 2, p = 0.26). In region B, all four locations were positive for DWV with significant variation between locations (location 2: 41.2% (7/17); location 3: 33.3% (1/3); location 4: 3.7% (1/27) and location 5: 2.3% (1/44); Pearson Chi Square test, χ^2^ = 22.3, df = 3, p<0.001). In region C, DWV was common in location 8 (77.7% (7/9)), but not found in locations 6, 7 and 9. Likewise, the samples of adult workers, sealed drone brood and mites of region A were also tested negative for DWV (N = 15 colonies).

#### Chronic bee paralysis virus

This virus was the least prevalent one as it was only identified in 5.96% (9/151) of all tested colonies. The majority of the CBPV-positive workers were found in region B. However, no significant differences were found between regions (Pearson Chi Square test, χ^2^ = 3.4, df = 2, p = 0.19). Locations 3, 4 and 5 were positive 33.3% (1/3), 15% (4/27) and 7% (3/44), respectively; but location 2 was free of this virus. Three out of four locations of the region C were negative and only location 9 was positive with 8.3% (1/12). The region A was also free of this virus.

Summarizing the prevalence of viral infections by regions, the SBV was 27%, 96.7% and 42.2% in regions A, B and C respectively; BQCV 72.5%, 20%, in the B,C regions, but in the region A that virus infection was not detected. Less common viruses DWV and CBPV were with mean prevalence from samples of B and C regions respectively 10.9 and 17.7; 8.7 and 2.2% (Table 1).

#### Multiple virus infections

The occurrence of multiple virus infections in the same regions was observed. Samples from the region B were infected with three to four viruses. Location 2 contained three virus infections and the locations 3, 4 and 5 contained all (four) detected viruses of this survey (Table 1). As shown in Table 1, adult bee worker samples in two locations (6 and 7) of the region C were contained single virus infection as only SBV. The location 8 contained three virus infections and location 9 two. Region A had only one virus infection (SBV).

Altogether, the following combined virus infections were observed: single virus occurrence contained only SBV; in dual infection-SBV and DWV; in triple-BQCV, SBV and DWV; in quadruple all detected four viruses. Interestingly, those viruses were not observed in any other combinations. In addition, single hives contained a maximum of three viruses at the time in any of these combinations SBV-BQCV-DWV and SBV-BQCV-CBPV.

#### Phylogeny of the detected viruses

The nucleotide sequences from SBV, BQCV, DWV and CBPV confirmed the identity of those viruses with 97–99% similarity.

**Fig 2:**
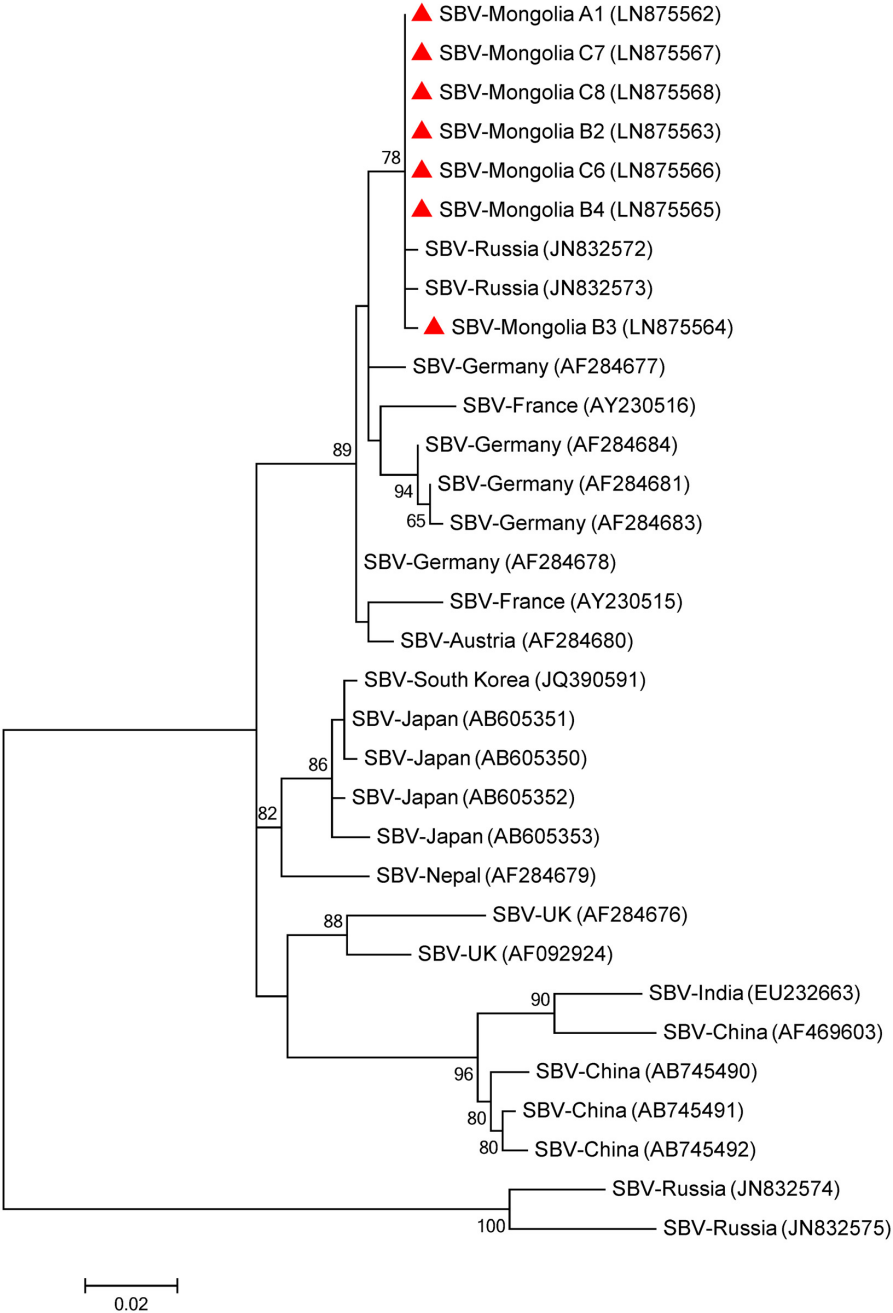
Phylogenetic tree of Sacbrood virus (SBV) isolates from Mongolia (red triangles) and other countries. The 541 nucleotide sequence of the partial RNA-dependent RNA polymerase gene from the three sampled regions and isolates from other countries were aligned by the MUSCLE program. The phylogenetic tree was constructed using the maximum likelihood method under the Tamura 3-parameter. Sequences obtained from GenBank are denoted with their country of origin and accession numbers. Bootstrap values >50 are shown in the corresponding nodes. The bar indicates the genetic distance scale (number of nucleotide differences per site).

**Fig 3:**
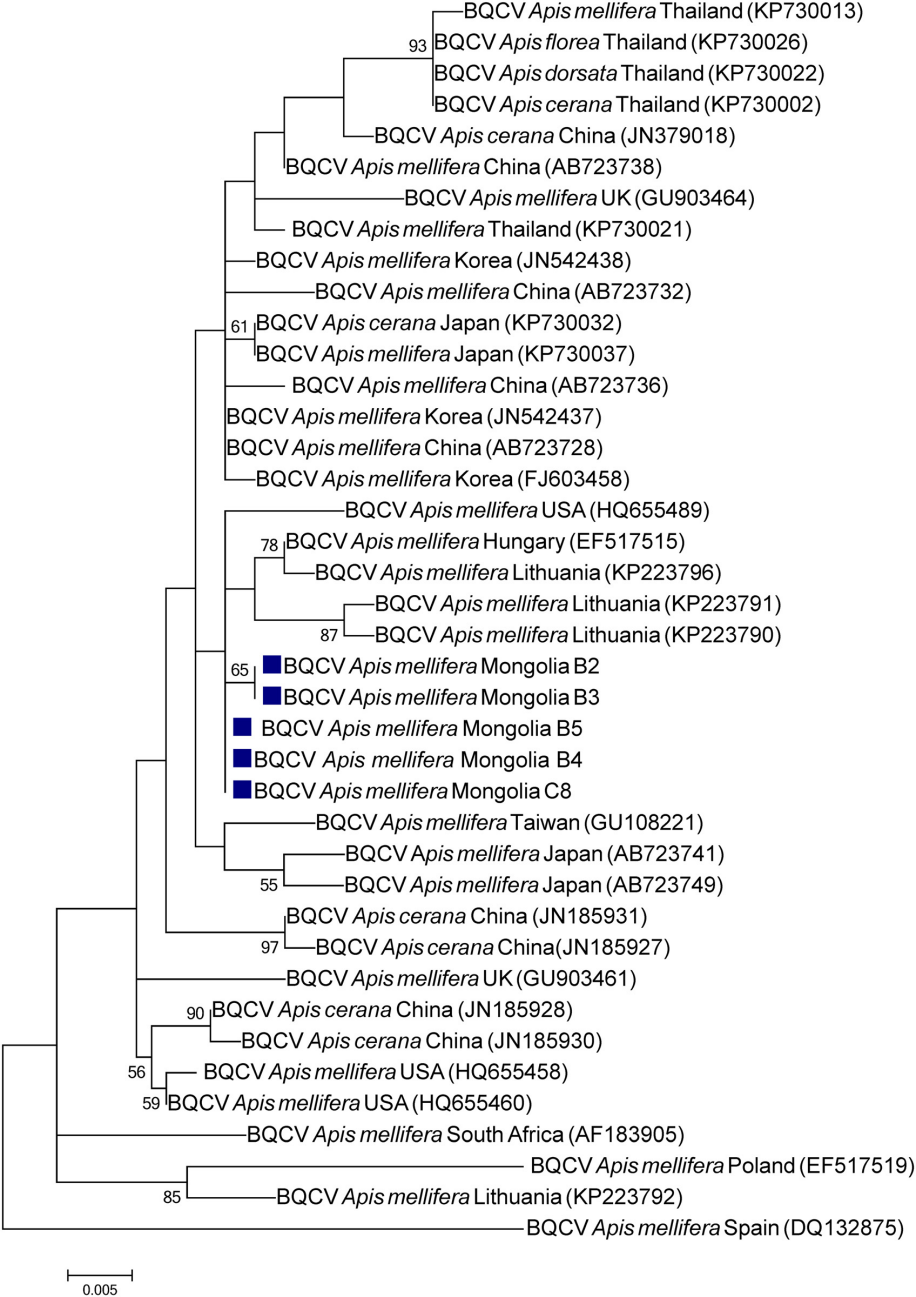
Phylogenetic tree of Black queen cell virus (BQCV) isolates from Mongolia (blue squares) and other countries. The 648 nucleotide sequence of partial capsid protein-coding region from two sampled regions and isolates from other countries were aligned by the MUSCLE program. The phylogenetic tree was constructed using the maximum likelihood method under the Tamura 3-parameter. Sequences obtained from GenBank are denoted with their country of origin, species host and accession numbers. Bootstrap values >50 are shown in the corresponding nodes. The bar indicates the genetic distance scale (number of nucleotide differences per site).

**Fig 4:**
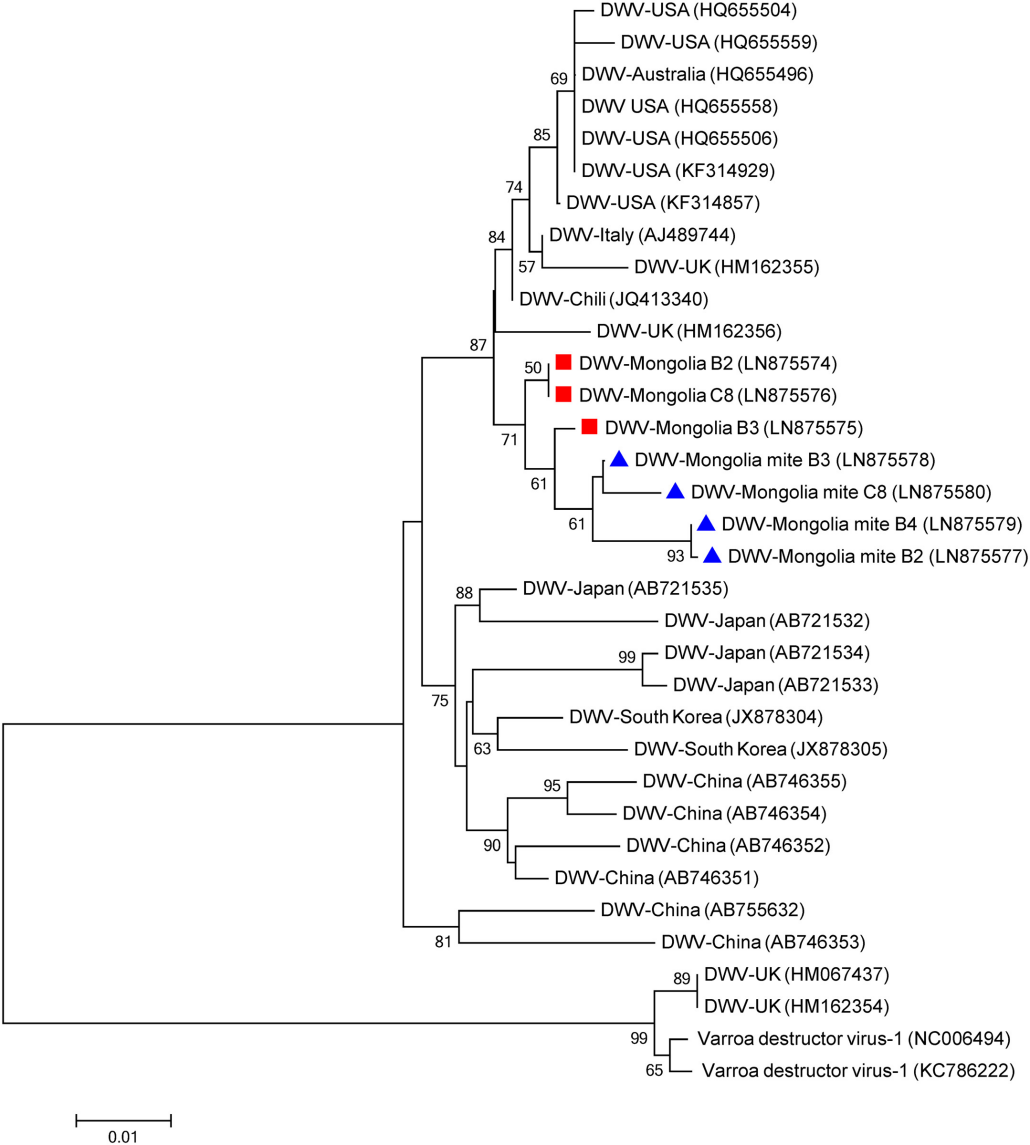
Phylogenetic tree of Deformed wing virus (DWV) isolates from honeybees (red squares) and *Varroa destructor* mites (blue triangles) from Mongolia. The 820 nucleotide sequence of partial capsid protein-coding region from two sampled regions and isolates from other countries were aligned by the MUSCLE program. The phylogenetic tree was constructed using the maximum likelihood method under the Tamura 3-parameter. Sequences obtained from GenBank are denoted with their country of origin and accession numbers. Bootstrap values >50 are shown in the corresponding nodes. Isolates from Varroa destructor virus-1 were used to root the tree. The bar indicates the genetic distance scale (number of nucleotide differences per site).

**Fig 5:**
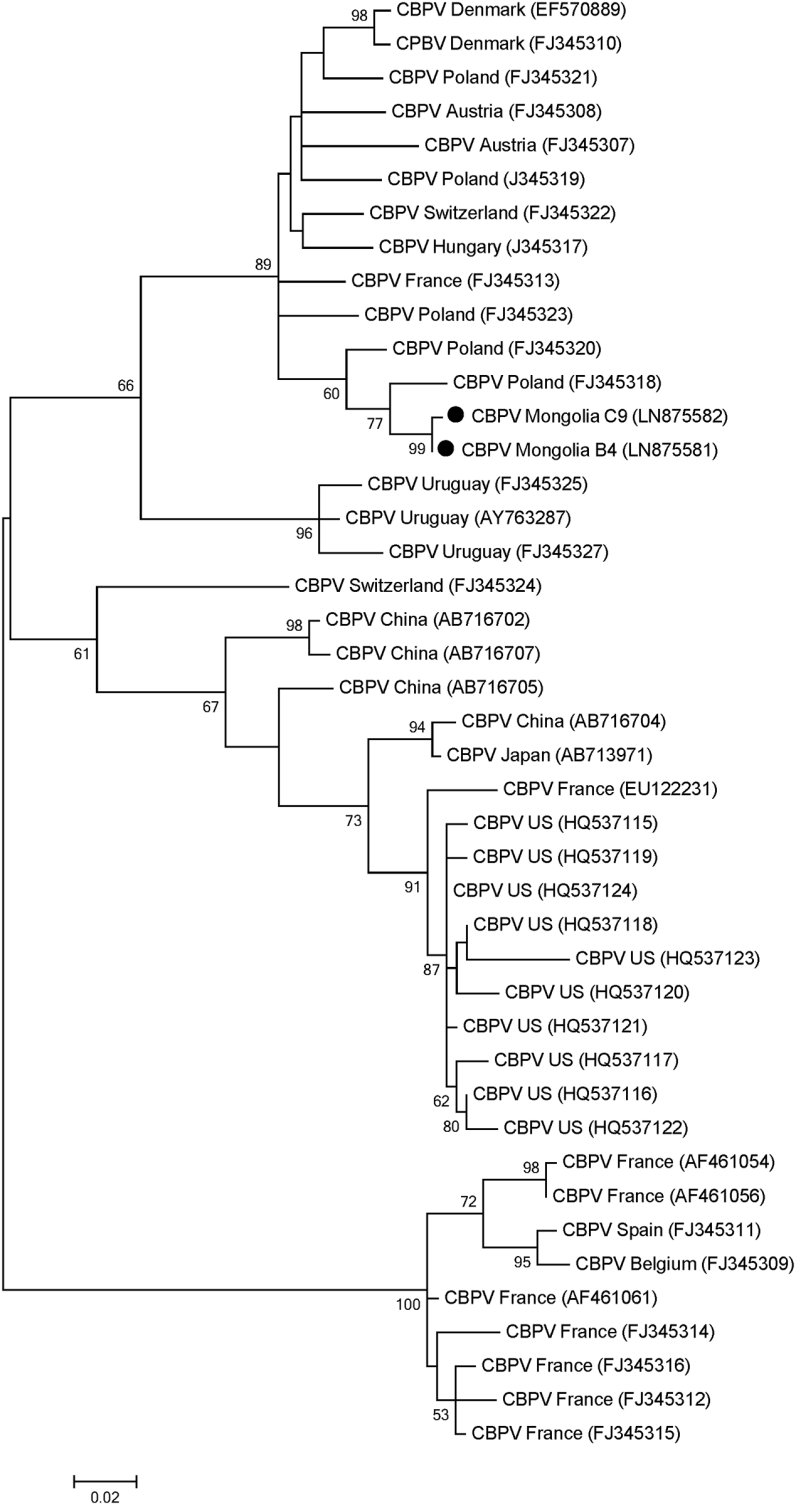
Phylogenetic tree of Chronic bee paralysis virus (CBPV) isolates from Mongolia (black circles) and other countries. The 335 nucleotide sequence of partial RdRp protein-coding region from two sampled regions and isolates from other countries were aligned by the MUSCLE program. The phylogenetic tree was constructed using the maximum likelihood method under the Tamura 3-parameter. Sequences obtained from GenBank are denoted with their country of origin and accession numbers. Bootstrap values >50 are shown in the corresponding nodes. The bar indicates the genetic distance scale (number of nucleotide differences per site.

Phylogenetic trees were inferred for those viruses (Figs 2–5). For the Mongolian SBV isolates, the tree was inferred from a 541-nt sequence of the RNA-dependent RNA polymerase gene (RdRp). Seven isolates from all three regions were used to construct the tree as well with previous isolates from Asia and Europe that were retrieved from Genbank (Fig 2). Most bootstrap values of the nodes are relatively high (>85%) supporting the isolates distribution in the tree. Five main clusters are observed in the tree. One cluster is conformed for isolates collected from Russia and continental European countries (Germany, Austria and France). A separated cluster is formed with UK isolates. Some Russian isolates also formed a separated cluster. The last two clusters are formed with Asian countries, one formed with Japanese and Korean isolates and the other formed with Chinese and Indian isolates. The tree indicates that the Mongolian isolates are closer related (89% bootstrap value) to isolates from Russia with European origin [27] and other isolates from Europe (Germany, Austria and France) than to Asian ones.

To infer the BQCV phylogenetic tree the 511-nt sequence of the capsid protein coding region was used. Five BQCV Mongolian isolates from the North and Central regions as well as sequences from isolates from USA, Europe and Asia were used to construct the tree (Fig 3). In general, the isolates cluster according to their geographical area of collection. It suggests that BQCV have some degree of geographical isolation. However, the low bootstrap values do not allow specifying similarities with previous isolates from other countries/continents.

The phylogeny of the Mongolian DWV isolates was inferred from 820-nt sequence of the capsid protein-coding region. Seven isolates from bees and mites from the North and Central regions as well as previous isolates retrieved from Genbank were used to construct the DWV phylogenetic tree (Fig 4). Two clusters are very well differentiated, one grouping the Asian isolates (South Korea, China and Japan) and the one grouping the isolates from USA, Australia, Chili, Italy and the UK. Also in this case, the Mongolian isolates cluster together in a monophyletic group showing more similarity with the later cluster (87% bootstrap value) than the one formed with Asian isolates.

To infer the CBPV phylogenetic tree the 335-nt sequence of the RdRp coding region was used. Two isolates derived from the North and Central regions together with isolates from other countries retrieved from the Genbank were used to construct the CBPV phylogenetic tree (Fig 5). Most bootstrap values of the nodes are relative high (>50%) supporting the isolates distribution in the tree. Similar to the other detected viruses, the two Mongolian isolates cluster together with European isolates with high bootstrap value (89%) showing similarities with isolates from Poland and clearly separated from the Asian isolates.

#### Relationships between observed pathogens

To reveal potential positive associations of pathogens in the samples from Mongolia, Pearson’s Chi-squared tests were performed (p<0.05). Overall locations, a positive correlation was found between the prevalence of *V*. *destructor* mites and DWV (Pearson r = 0.704, df = 7, two tailed P = 0.03) especially in region B and C (r = 0.801 and 0.893, respectively). No other significant correlations were found.

## Discussion

This is the first report addressing the honey bee, *Apis mellifera*, pathosphere of Mongolia. The survey data show a distinctive pathosphere of colonies of European, *A*. *mellifera*, subspecies in this isolated country, where honey bees have been introduced as recent as ~60 years ago. We used standard methods to survey 151 colonies, distributed in three regions and 9 locations across the country, to register the presence of 14 parasites and pathogens of managed honey bees. Besides the mite *V*. *destructor*, the sampling scheme allowed for the detection of the tested pathogens, when only 5% of workers are infested with a probability of 0.92 [28]. Therefore, our detection power was sufficient to identify even colonies with low pathogen prevalence. However, these results represent the pathosphere only during the summer season and caution has to be taken as some pathogens are driven by strong seasonality, e.g. DWV usually has a peak in autumn [29].

Despite this high detection power, the causative bacteria for American and European foulbrood (*P*. *larvae* and *M*. *plutonius*, respectively) were not detected in any of the sampled colonies, thereby indicating their absence or most probably a very low prevalence at the time of sampling. AFB and EFB are widely distributed around the globe [5, 12, 30], but their prevalence is characterized by a high spatial-temporal variation. For instance, a recent systematic survey across 17 European countries [31] found AFB in most countries with the exception of Belgium, Germany and UK, where AFB has previously been reported [32–34]. Similarly, the tracheal mite, *A*. *woodi*, was not detected. Although this mite is thought to have a global distribution, it has also not been found in Sweden, Norway, Denmark, New Zealand, and Australia, and in the US state of Hawaii [35].

Since the first report of *N*. *ceranae* in Asia [36] and its subsequent expansion into Europe [37], it seems most likely that this novel microsporidian has gradually been replacing the previously established *N*. *apis* [38]. To our knowledge, there is no prior record of *N*. *apis* in Mongolia. Therefore, the absence of *N*. *apis* in our samples may indicate a complete takeover of the *A*. *mellifera* host by *N*. *ceranae* in this country. Alternatively, *N*. *apis* was absent from this isolated region prior to the arrival of *N*. *ceranae*. Interestingly, there was no significant correlation between the prevalence of *N*. *ceranae* and BQCV as previously been described for *N*. *apis* and this virus [39]. *N*. *ceranae* was restricted to region B with a prevalence of 20.88% (19/91), thereby suggesting that this region had some contact with bees or beekeeping material infested/contaminated with respective spores. The prevalence of *N*. *ceranae* in all surveyed regions in Mongolia was 12.58% (19/151). This is considerably high in light of a recent European survey that included 16 countries, and showed that only 3 countries (Hungary, Poland and Sweden) exceeded 10% of clinical prevalence [31]. The underlying reasons for the absence of *N*. *ceranae* in regions A and C remain unclear, but the extreme isolation of some apiaries (up to 600 km) cannot be overruled. *N*. *ceranae* is expected to be affected by extreme cold climate [40], but this apparently did not limit high infestation levels in Mongolia as well as in other countries with similar winter conditions (e.g. Sweden) [31].

The prevalence of *V*. *destructor* in the Mongolian apiaries varied between the geographical locations from none (location 7, 0/15) to all (location 8, 9/9) of the colonies infested. Those variations may be explained by differential mite treatments, e.g. in region B and location 7. In general, considerable differences in mite infestation levels between countries, regions or even apiaries are not uncommon. For example, samples collected at the same period of time (summer 2013) can show substantial differences ranging from 0% (Belgium) to 87% (Latvia) at the apiary level [31].

At present, there are no data about colony losses in Mongolia to link possible effect of mite infestation on the health of local colonies. Interestingly, despite a significant positive correlation between the prevalence of DWV and *V*. *destructor* overall surveyed regions, DWV and *V*. *destructor* were not always found together at the location level. For example in locations 1, 6 and 9 only *V*. *destructor* was found but not DWV. In location 7, neither DWV nor *V*. *destructor* were found, possibly due to treatments by beekeepers. Mongolia hence offers a unique opportunity to address *V*. *destructor* and DWV in isolation. In sharp contrast to other areas [22,41–45], DWV was comparatively rare compared to other viruses.

Regarding viruses in general, SBV was the most prevalent one in our surveyed colonies (73.5%). This high prevalence of SBV is in line with other studies from European and Asian countries (64% France, [42]; 40% Croatia, [43]; 19% Belgium, [46]; 39% Japan, [22]; 21% China, [47]; 5% Czech Republic, [44]). BQCV was the second most prevalent virus in our colonies (49.7%). It has been reported with even higher frequencies in Japan and France (77% [22], and 58% [42], respectively) and with lower frequencies in China, Croatia, Czech Republic and Belgium [41,43–44,46]. Surprisingly, the prevalence of DWV (11.3%) was also low in comparison with other studies [22,41–45], despite the regular presence of the *V*. *destructor* mite, which is acting as a potent vector of this virus [48]. The prevalence of CBPV was low (5.9%) in concordance with other studies [41–45]. LSV 2, ABPV, KBV and IAPV were not detected in this survey although they have been found in a range of other studies in Europe and Asia [22,41–42,45–46].

Since the prevalence of some viruses (e.g. DWV, IAPV) and parasites (e.g. *Nosema* spp.) can be governed by season [29,31,42], the present sampling scheme limited to summer only may have underestimated the occurrence of some pathogens. The region B was the most infected one with all four detected viruses being present there. This may indicate that the transmission of viruses between local colonies may be fostered, e.g. by beekeeping practices as the sharing of material and movement of colonies.

The phylogenetic trees indicate that the detected viruses are more related to European strains than to Asian ones suggesting that the Mongolian apiaries are isolated from South Asia. Despite the relative close distance to those countries, the Gobi desert at the south of the country as well as various high mountains (e.g. the Altai) along the border with China impose natural geographic barriers, which seem to efficiently limit contact between honey bees from Mongolia and those from South-Asian countries. This may explain the distant phylogenetic relation between those geographically closed populations. The imports of honey bee colonies from Russia are the most plausible source of the detected European viruses, thereby supporting the view that global apicultural trade was and still is the most efficient way for long range transport of pathogens.

In conclusion, this survey reveals to a certain level, the distinctive honey bee, *A*. *mellifera*, pathosphere of Mongolia, with the absence or low prevalence of otherwise globally distributed diseases, e.g. the presence of *V*. *destructor* in isolation from DWV. This creates an interesting case, which offers ample opportunity for exciting future research on honey bee health.

## Supporting Information

S1 Table Used primer sets for the molecular detection of pathogens. (S) indicates the primer sets used for sequencing.
(DOCX)
