# Supplementary material for: The Honey Bee Pathosphere of Mongolia: European Viruses in Central Asia
Source: PLoS One. 2016 Mar 9;11(3):e0151164. doi: 10.1371/journal.pone.0151164 (PMC4784942; doi:10.1371/journal.pone.0151164)
Supplement: S1 Table — (S) indicates the primer sets used for sequencing. (DOCX) [file pone.0151164.s001.docx]

**Supporting information**

**S1 Table.-** Used primer sets for the molecular detection of pathogens. (S) indicates the primer sets used for sequencing.

| **Pathogen** | **Sequence of primer set** | **Size (bp)** | **Reference** |  |
| --- | --- | --- | --- | --- |
| *Varroa destructor* (S) | 5’ CTTGTAATCATAAGGATATTGGAAC 3’(F)  5’ AATACCAGTGGGAACCGC 3’ (R) | 929 | Navajas et al. 2010 |  |
| *Nosema apis* | 5’ CCATTGCCGGATAAGAGAGT 3’ (F)  5’ CACGCATTGCTGCATCATTGAC 3’ (R) | 268 | Chen et al., 2008 |  |
| *Nosema ceranae* | 5’ CGGATAAAAGAGTCCGTTACC 3’ (F)  5’ TGAGCAGGGTTCTAGGGAT 3’(R) | 250 | Chen et al., 2008 |  |
| *Acarapis woodi* | 5’ TCTTCAATTTTAATTATACGT 3’ (F)  5’ AAAAATCAGAATAAATGTTGAAATA 3’ (R) | 220 | Kojima et al., 2011 |  |
| *Melissococcus plutonius* | 5’ACTGAAACAATGCATTTGCAC 3’ (F) 5’AGTGGTGAATCTTGGTTGGCT 3’ (R) | 401 | Gauthier et al. unpublished |  |
| *Paenibacillus larvae* | 5’ GCAAGTCGAGCGGACCTTGT 3’(F)  5’ AGAAGAGACTTCAAGGAC3’ (R) | 438 | Gauthier et al. unpublished |  |
| ABPV | 5’ TCATACCTGCCGATCAAG 3’ (F)  5’ CTGAATAATACTGTGCGTATC 3’ (R) | 197 | de Miranda et al., 2010 |  |
| BQCV | 5’ AGTGGCGGAGATGTATGC 3’ (F)  5’ GGAGGTGAAGTGGCTATATC 3’ (R) | 294 | Locke et al., 2012 |  |
| BQCV (S) | 5’ GTGGCGGAGATGTATGCGCTTTATC 3’ (F)  5’ CTGACTCTACACACGGTTCGATTAG 3’ (R) | 511 | Yang et al., 2013 |  |
| CBPV | 5’ CAACCTGCCTCAACACAG 3’ (F)  5’ AATCTGGCAAGGTTGACTGG 3’ (R) | 296 | Locke et al., 2012 |  |
| CBPV (S) | 5’ TAYGAGYGATTTYTTGRGATCGAYTTCGCT 3’ (F)  5’ TGTAYTCGRCCTGATTRACGACRTTAGC 3’ (R) | 335 | Yang et al., 2013 |  |
| DWV | 5’ CGTCGGCCTATCAAAG 3’ (F)  5’ CTTTTCTAATTCAACTTCACC 3’ (R) | 417 | Yañez et al., 2012 |  |
| DWV (S) | 5’ GCGAGCCAAATCAGGGCAAAACCTG 3’ (F)  5’ GGCGCGACCAAATCCACTCGACTGT 3’ (R) | 820 | Yang et al., 2013 |  |
| IAPV | 5’ CCATGCCTGGCGATTCAC 3’ (F)  5’ CTGAATAATACTGTGCGTATC 3’ (R) | 203 | de Miranda et al., 2010 |  |
| KBV | 5’ CCATACCTGCTGATAACC 3’ (F)  5’ CTGAATAATACTGTGCGTATC 3’ (R) | 200 | de Miranda et al., 2010 |  |
| SBV | 5’ TTGGAACTACGCATTCTCTG 3’ (F)  5’ GCTCTAACCTCGCATCAAC 3’ (R) | 335 | Locke et al., 2012 |  |
| SBV (S) | 5’ AATGGTGCGGTGGACWATGGRGCAYGT 3’ (F)  5’ TGATACAGRGCRGCTCGRCARTTYTC 3’ (R) | 558 | Yang et al., 2013 |  |
| LSV (Strain 2) | 5’ CGTGCTGAGGCCACGGTTGT 3’ (F)  5’ GCGGTGTCGATCTCGCGGAC 3’ (R) | 226 | Runckel *et al.* 2011 |  |
|  | | | | |

- Chen Y, Evans JD, Smith IB, Pettis JS. *Nosema ceranae* is a long-present and wide-spread microsporidian infection of the European honey bee (*Apis mellifera*) in the United States. J Invertebr Pathol. 2008;97(2):186-8.
- de Miranda JR, Cordoni G, Budge G. The acute bee paralysis virus–Kashmir bee virus–Israeli acute paralysis virus complex. J Invertebr Pathol. 2010;103, Supplement:S30-S47.
- Kojima Y, Toki T, Morimoto T, Yoshiyama M, Kimura K, Kadowaki T. Infestation of Japanese Native Honey Bees by Tracheal Mite and Virus from Non-native European Honey Bees in Japan. Microb Ecol. 2011;62(4):895-906.
- Locke B, Forsgren E, Fries I, de Miranda JR. Acaricide treatment affects viral dynamics in *Varroa destructor*-infested honey bee colonies via both host physiology and mite control. J Appl Environ Microbiol. 2012;78(1):227-35.
- Navajas M, Anderson DL, de Guzman LI, Huang ZY, Clement J, Zhou T, et al. New Asian types of *Varroa destructor*: a potential new threat for world apiculture. Apidologie. 2010;41(2):181-93.
- Runckel C, Flenniken ML, Engel JC, Ruby JG, Ganem D, Andino R, et al. Temporal Analysis of the Honey Bee Microbiome Reveals Four Novel Viruses and Seasonal Prevalence of Known Viruses, *Nosema*, and *Crithidia*. PLoS ONE. 2011;6(6):e20656.
- Yang B, Peng GD, Li TB, Kadowaki T. Molecular and phylogenetic characterization of honey bee viruses, Nosema microsporidia, protozoan parasites, and parasitic mites in China. Ecol Evol. 2013;3(2):298-311.
- Yañez O, Jaffé R, Jarosch A, Fries I, Moritz RA, Paxton R, et al. Deformed wing virus and drone mating flights in the honey bee (*Apis mellifera*): implications for sexual transmission of a major honey bee virus. Apidologie. 2012;43(1):17-30.
